# Effects of icariside II on brain tissue oxidative stress and Nrf2/HO-1 expression in rats with cerebral ischemia-reperfusion injury^1^

**DOI:** 10.1590/s0102-8650201900208

**Published:** 2019-02-28

**Authors:** Yan Li, Fanjun Meng

**Affiliations:** I MD, Department of Anesthesiology, Jinan City Central Hospital Affiliated to Shandong University, P.R. China. Technical procedures, acquisition of data, statistics analysis, critical revision, final approval.; II MD, Department of Anesthesiology, Jinan City Central Hospital Affiliated to Shandong University, P.R. China. Design of the study, manuscript preparation, critical revision, final approval.

**Keywords:** Icariside II, Reperfusion Injury, Brain Ischemia, Oxidative Stress, Rats

## Abstract

**Purpose:**

To investigate the effects of icariside II on brain tissue oxidative stress and Nrf2/HO-1 expression in rats with cerebral ischemia-reperfusion injury (CIRI).

**Methods:**

One hundred SD rats were randomly divided into sham-operated, model, and 5, 10 and 20 mg/kg icariside II groups, 20 rats in each group. The middle cerebral artery occlusion model (ischemia for 2 h followed by reperfusion for 24 h) was established in the later 4 groups. In later 3 groups, at reperfusion beginning, the rats were intragastrically administrated with 5, 10 and 20 mg/kg icariside II, respectively. After 24 h of reperfusion, the neurological severity score, cerebral water content and cerebral infarction volume, brain tissue oxidative stress indexes and Nrf2 and HO-1 protein expressions were determined.

**Results:**

Compared with model group, in 20 mg/kg icariside II group the neurological severity score, cerebral water content and cerebral infarction volume, brain tissue ROS content and MDA level were significantly decreased (P<0.05), and the brain tissue SOD, GSH-Px and catalase levels and Nrf2 and HO-1 protein levels were significantly increased (P<0.05).

**Conclusion:**

Icariside II can alleviate the CIRI in rats through reducing brain tissue oxidative stress and improving Nrf2/HO-1 expression.

## Introduction

**Figure 1:**
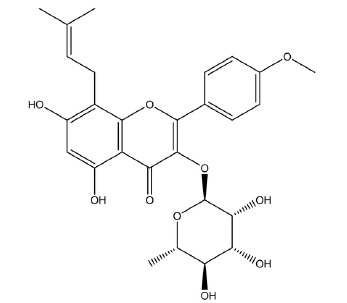
Chemical structure of icariside II.

 Cerebral ischemia reperfusion injury (CIRI) refers to the more serious injury and dysfunction of ischemic brain tissue after restoring the blood perfusion, compared with before perfusion. The mechanism of CIRI includes several aspects, in which the oxidative stress and cytotoxic injury caused by endogenous and exogenous electrophiles are one of the important mechanisms^1^. After cerebral ischemia, the production of reactive oxygen species (ROS) in cells is increased sharply, thus leading to the oxidative stress^2^. In traumatic brain injury, Nrf2/HO-1 pathway has been found to be one of the most important antioxidant and cytotoxic defense mechanisms in cells. Up-regulation of this signaling pathway can induce a variety of antioxidant enzymes and detoxifying enzymes, accelerate the enzymatic reaction, increase the expression of superoxide dismutase (SOD), glutathione and other antioxidant substances, scavenge free radicals and other oxides, and maintain the normal intracellular potential level, thus playing a role in cell protection^3^ ^,^ ^4^. *Herba Epimedii* is a Berberidacae medicinal plant in Asia. Icariside II (5,7-dihydroxy-2-(4-methoxyphenyl)-8-(3-methylbut-2-enyl)-3-[(2S,3R,4R,5R,6S)-3,4,5-trihydroxy-6-methyloxan-2-yloxychromen-4-one) is one of the main active ingredients of *Herba Epimedii* (Fig. 1). 

 Modern pharmacological studies have proved that icariside II has multiple pharmacological effects such as improving cardiovascular and cerebrovascular function, anti-cancer, anti-oxidation, anti-osteoporosis and delaying aging^5^ ^-^ ^9^. However, the effects of icariside II on CIRI are seldom reported. In the present study, we investigated the effects of icariside II on oxidative stress and Nrf2/HO-1 expression in rats with CIRI, for providing an experimental reference for clinical application of icariside II to prevention and treatment of CIRI.

## Methods

 This study was approved by the ethics committee of the Jinan City Central Hospital Affiliated to Shandong University. All animal procedures followed the Principles of Laboratory Animal Care and were in accordance with the Guide for the Care and Use of Laboratory Animals by the National Institutes of Health.

 A total of 100 healthy male SD rats weighing 250-300 g were adaptively raised for 7 days, with freely eating and drinking. The room temperature was 25±2^o^C, with 12h-light/12h-dark cycle illumination. The rats were randomly divided into sham-operated group, model group, 5 mg/kg icariside II group 10 mg/kg icariside II group and 20 mg/kg icariside II group, 20 rats in each group. The rats were fasted for 12 h before modeling.

###  Animal modeling and treatment 

 The middle cerebral artery occlusion model of rats was established according to the method reported by Longa *et al.* ^10^. After anesthesia by intraperitoneal injection of 10% chloral hydrate, the rats were fixed in supine position. The skin of the median neck was incised, and the left external carotid artery and internal carotid artery were separated. The main external carotid artery was ligated and freed. A small incision was made in the external carotid artery. The nylon thread was inserted into the internal carotid artery through the external carotid artery, with depth of 18.5±0.5 mm, and the blood supply of the middle cerebral artery was blocked for ischemia for 2 h. Then, the thread was gently pulled out for the blood flow reperfusion for 24 h. In the sham-operated group, the procedure was the same with other groups excepting inserting the nylon thread. In 5, 10 and 20 mg/kg icariside II groups, after ischemia for 2 h (at reperfusion beginning), the rats were aintragastrically administrated with 5, 10 and 20 mg/kg icariside II (purity 95%), respectively. The sham-operated and model groups were synchronously aintragastrically administrated with equal volume of normal saline.

###  Neurological severity scoring 

 After 24 h of reperfusion, the neurological severity of rats was scored using the classical Zea Longa method^10^ as follows: 0 point: no symptom of neurological deficit was observed; 1 point: the front claw on the operation-opposite side could not extend, and the rats could limp (slight neurological deficit); 2 points: the rats could not go straight, only walking in circles towards the operation-opposite side (moderate neurological deficit); 3 points: the rats tilted towards the operation side (severe neurological deficit); 4 points: the rats were unable to walk, with loss of consciousness.

###  Determination of cerebral water content 

 According to the reported method^11^, 5 rats in each group were taken. After intraperitoneal injection of uratan for anesthesia, the brain of rats was cut off, and the brain tissues were stripped. After removal of olfactory bulb, cerebellum and low brainstem, the brain tissues were weighted, and the wet mass was obtained. Then, the brain tissues were placed in 110^o^C constant-temperature drying oven for 48 h. After weighting, the dry mass was obtained. The cerebral water content was calculated as follows: cerebral water content (%) = (wet mass - dry mass) / wet mass × 100.

###  Determination of cerebral infarction volume 

 According to the reported method^12^, 5 rats in each group were taken. After intraperitoneal injection of uratan for anesthesia, the brain of rats was cut off, and the coronal sections were made. The sections were incubated in 2% 2,3,5-triphenyltetrazolium chloride staining solution for 30 min under constant temperature. The red area presented the normal brain tissue, and the pale area presented the infarcted brain tissue. The cerebral infarction volume (presented by percentage) was analyzed by Imagepro Plus 6.0 image analysis software.

###  Determination of oxidative stress indexes in brain tissue 

 Five rats in each group were taken. After intraperitoneal injection of uratan for anesthesia, the brain of rats was taken. After homogenizing, the brain tissues were centrifuged at 3000 r/min for 10 min, the supernatant was obtained. The ROS content in brain tissue was determined using ROS enzyme-linked immunosorbent assay kits. The oxidative stress indexes including SOD, glutathione peroxidase (GSH-Px), catalase and malondialdehyde (MDA) in brain tissues were measured by ultraviolet-visible spectrophotometer. 

###  Determination of Nrf2 and HO-1 protein expression in brain tissue 

 Five rats in each group were taken. After intraperitoneal injection of uratan for anesthesia, the brain of rats was taken. The whole cell, nucleus and cytoplasmic protein were extracted according to the instructions of kits, and the protein content was determined. The protein samples were mixed with SDS gel buffer and boiled to denaturation. After SDS-PAGE electrophoresis, the protein was separated and transferred to PVDF membrane, followed by blocking with TBS solution containing 5% skim milk powder. Then the membranes were incubated with rabbit anti-mouse β-actin antibody (1: 1000), rabbit anti-mouse Nrf2 antibody (1: 1000), rabbit anti-mouse HO-1 antibody (1: 500) overnight at 4^o^C, followed by washing with TBST solution for three times. Then, the membranes were incubated with HRP-labeled secondary antibody (1: 1000) at room temperature, followed by developing with an enhanced HRP-DAB substrate chromogenic kit. The membranes were scanned and the integrated optical density of the target band was determined by image analysis software. The ratio of integrated optical density value of target protein band to β-actin band was used as the relative expression of target protein. The kits were provided by Fuzhou Maixin Biotechnology Development Co., Ltd. (Fuzhou, China).

###  Statistical analysis 

 Data were presented as mean±SD, and analyzed using SPSS 20.0 software (SPSS Inc., Chicago, IL, USA). The comparisons among different groups were performed using single-factor analysis of variance followed by Dunnett’s multiple-comparisons post-hoc test. P < 0.05 was considered as statistically significant.

## Results

###  Neurological severity score 

**Figure 2:**
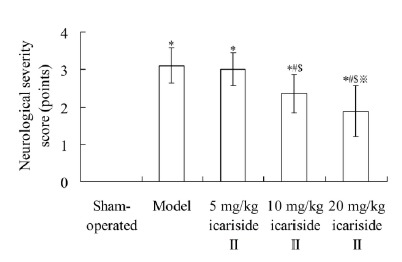
Effect of icariside II on neurological severity score. Values are mean±SD. ^*^P < 0.05 *vs* sham-operated group; ^#^P < 0.05 *vs* model group; ^$^P < 0.05 *vs* 5 mg/kg icariside II group; ^※^P < 0.05 *vs* 10 mg/kg icariside II group.

 As shown in Fig. 2, the neurological severity score in model group was 3.11±0.47 points, which was significantly higher than 0.00±0.00 point in sham-operated group (P < 0.05). The neurological severity score in 10 mg/kg icariside II and 20 mg/kg icariside II groups was 2.36±0.52 points and 1.88±0.68 points, respectively, which was significantly lower than that in model group, respectively (P < 0.05). 

###  Cerebral water content and cerebral infarction volume 

Table 1 showed that, compared with sham-operated group, in model, 5 mg/kg icariside II, 10 mg/kg icariside II and 20 mg/kg icariside II groups the cerebral water content and cerebral infarction volume were significantly increased, respectively (P < 0.05). Compared with model group, the cerebral water content and cerebral infarction volume in 10 mg/kg icariside II and 20 mg/kg icariside II groups were significantly decreased, respectively (P < 0.05).

**Table 1:** Effect of icariside II on cerebral water content and infarction volume.

Group	Cerebral water content (%)	Cerebral infarction volume (%)
Sham-operated	76.33±1.42	0.00±0.00
Model	80.12±1.92^*^	26.34±3.12^*^
5 mg/kg icariside II	79.88±1.24^*^	25.52±2.55^*^
10 mg/kg icariside II	78.88±1.55^*#^	20.88±2.15^*#$^
20 mg/kg icariside II	77.52±1.23^*#$^	16.36±2.09^*#$※^

Values are mean±SD. ^*^P < 0.05 *vs* sham-operated group; ^#^P < 0.05 *vs* model group; ^$^P < 0.05 *vs* 5 mg/kg icariside II group; ^※^P < 0.05 *vs* 10 mg/kg icariside II group.

###  ROS content in brain tissue 

**Figure 3:**
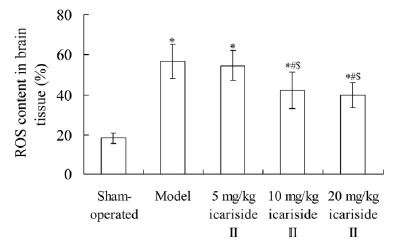
Effect of icariside II on ROS content in brain tissue. Values are mean±SD. ^*^P < 0.05 *vs* sham-operated group; ^#^P < 0.05 *vs* model group; ^$^P < 0.05 *vs* 5 mg/kg icariside II group. ROS, reactive oxygen species.

 As shown in Fig. 3, compared with sham-operated group, in model, 5 mg/kg icariside II, 10 mg/kg icariside II and 20 mg/kg icariside II groups the ROS content in brain tissue was significantly increased, respectively (P < 0.05). Compared with model group, the ROS content in 10 mg/kg icariside II and 20 mg/kg icariside II groups was significantly decreased, respectively (P < 0.05).

###  SOD, GSH-Px, catalase and MDA levels in brain tissue 

 Compared with sham-operated group, in model, 5 mg/kg icariside II, 10 mg/kg icariside II and 20 mg/kg icariside II groups the SOD, GSH-Px and catalase levels in brain tissue were significantly decreased, respectively (P < 0.05), and the MDA level in brain tissue was significantly increased (P < 0.05). Compared with model group, the SOD level in 20 mg/kg icariside II group and GSH-Px and catalase levels in 10 and 20 mg/kg icariside II groups were significantly increased, respectively (P < 0.05), and the MDA level in 5, 10 and 20 mg/kg icariside II groups was significantly decreased, respectively (P < 0.05) (Table 2).

**Table 2:** Effect of icariside II on SOD, GSH-Px, catalase and MDA levels in brain tissue.

Group	SOD (U/mg)	GSH-Px (U/mg)	Catalase (U/mg)	MDA (nmol/mg)
Sham-operated	16.29±2.44	18.55±3.45	5.12±1.12	4.58±1.05
Model	8.95±2.02^*^	10.67±2.73^*^	2.27±0.34^*^	8.48±1.77^*^
5 mg/kg icariside II	9.34±2.15^*^	11.38±2.42^*^	2.58±0.47^*^	7.05±1.43^*#^
10 mg/kg icariside II	10.03±2.34^*^	12.31±2.83^*#^	3.17±0.59^*#^	6.33±1.31^*#$^
20 mg/kg icariside II	12.27±2.63^*#$^	14.39±2.94^*#$^	4.21±0.49^*#$※^	5.74±1.09^*#$※^

Values are mean±SD. ^*^P < 0.05 *vs* sham-operated group; ^#^P < 0.05 *vs* model group; ^$^P < 0.05 *vs* 5 mg/kg icariside II group; ^※^P < 0.05 *vs* 10 mg/kg icariside II group. SOD, superoxide dismutase; GSH-Px, glutathione peroxidase; MDA, malondialdehyde.

###  Nrf2 and HO-1 protein expressions in brain tissue 

 As shown in Table 3, compared with sham-operated group, the Nrf2 and HO-1 protein expression levels in model, 5 mg/kg icariside II, 10 mg/kg icariside II and 20 mg/kg icariside II groups were significantly increased, respectively (P < 0.05). Compared with model group, the Nrf2 protein level in 5, 10 and 20 mg/kg icariside II group and HO-1 protein level in 10 and 20 mg/kg icariside II groups were significantly increased, respectively (P < 0.05).

**Table 3:** Effect of icariside II on Nrf2 and HO-1 protein expressions in brain tissue.

Group	Nrf2/β-actin	HO-1/β-actin
Sham-operated	0.98±0.22	0.68±0.19
Model	1.52±0.29^*^	1.34±0.25^*^
5 mg/kg icariside II	1.94±0.27^*#^	1.46±0.29^*^
10 mg/kg icariside II	2.34±0.36^*#$^	1.95±0.34^*#$^
20 mg/kg icariside II	2.84±0.44^*#$※^	2.58±0.41^*#$※^

Values are mean±SD. ^*^P < 0.05 *vs* sham-operated group; ^#^P < 0.05 *vs* model group; ^$^P < 0.05 *vs* 5 mg/kg icariside II group; ^※^P < 0.05 *vs* 10 mg/kg icariside II group.

## Discussion

 In treatment of ischemic cerebrovascular diseases, restoring the blood flow in ischemic area or strengthening the blood supply to ischemic area is the prerequisite for alleviating the damage to the structure and function of central nervous system cells. However, if the dredging and restoring of cerebral blood flow exceed a certain time point, they cannot alleviate the tissue damage and dysfunction caused by ischemia, and even cause further aggravation of nerve injury, which is called CIRI^13^. Inhibiting the reperfusion injury is an important link in the treatment of ischemic cerebrovascular diseases. This study has established the CIRI model of rats, and investigated the effects of icariside II on the oxidative stress and Nrf2/HO-1 expression in CIRI rats. Results showed that, the neurological severity score, cerebral water content and cerebral infarction volume in model group were significantly higher than those in sham-operated group. Compared with model group, the neurological severity score, cerebral water content and cerebral infarction volume in 10 mg/kg icariside II and 20 mg/kg icariside II groups were significantly decreased, respectively. This indicates that, icariside II has the protective effects on CIRI in rats.

 Under the normal physiological conditions, the ROS generated in body can be reduced to hydrogen peroxide under the catalysis of SOD, and further reduced to harmless water and oxygen under the catalysis of GSH-Px or catalase, thus maintaining the dynamic balance of ROS in body^14^. SOD, GSH-Px and catalase together constitute the anti-oxidation defense system in body. Their activities can directly reflect the body’s antioxidant capacity. A large number of ROS produced during cerebral ischemia excessively consume the endogenous antioxidant enzymes, leading to the changes in the expression and activity of various enzymes such as SOD, GSH-Px, CAT, etc.^15^ The decreased activity of antioxidant enzymes reduces the ability of brain tissue in scavenging ROS. Excessive accumulation of ROS causes the lipid and protein oxidation, DNA damage, energy metabolism failure, and even the cell death^16^. MDA is the final product of lipid peroxidation induced by ROS attack on polyunsaturated fatty acids in biofilm. The change of MDA content indirectly reflects the change of ROS content and the degree of tissue damage^17^. Results of this study showed that, compared with sham-operated group, in model group the ROS content and MDA level in brain tissue were significantly increased, and the SOD, GSH-Px and catalase levels in brain tissue were significantly decreased. Compared with model group, the ROS content and MDA level in 10 mg/kg icariside II and 20 mg/kg icariside II groups were significantly decreased, and the SOD level in 20 mg/kg icariside II group and GSH-Px and catalase levels in 10 mg/kg icariside II and 20 mg/kg icariside II groups were significantly increased. This indicates that, icariside II has the ability of enhancing the antioxidant enzyme activities in brain tissue, scavenging the ROS and reducing the lipid peroxidation, thus playing a role in alleviating the CIRI.

 Nrf2 is an important transcription factor regulating antioxidant stress. Kelch-like ECH-associated protein 1 (Keap 1) is its specific receptor. Normally, a complex of Keap1 and Nrf2 is formed in the cytoplasm for inhibiting the activity of Nrf2. Under the oxidative stress, Keap1 dissociates with Nrf2, and Nrf2 translocates into the nucleus. Nrf2 binds to antioxidant response elements (ARE), activates ARE-regulated gene expression of phase II detoxifying enzymes and antioxidant enzymes, and enhances cell resistance to oxidative stress and nucleophilic compounds^18^. The antioxidant genes regulated by ARE include HO-1, glutathione-S-transferase and so on. These enzymes can protect the body from the damage of ROS^19^ ^,^ ^20^. It is confirmed that, the severity of cerebral ischemic injury is aggravated in Nrf2 gene-knockout mice^21^. In addition, activating the Keap1/Nrf2/ARE pathway and promoting the expression of HO-1 and other genes can alleviate the cerebral ischemic injury^22^. In the present study, compared with sham-operated group, in model group the Nrf2 and HO-1 protein expression levels in brain tissue were increased. This indicates that, the Nrf2/HO-1 signaling pathway in brain tissues is activated by oxidative stress. Compared with model group, the Nrf2 protein level in 5, 10 and 20 mg/kg icariside II group and HO-1 protein level in 10 and 20 mg/kg icariside II groups were significantly increased. This indicates that, icariside II can further activate the Nrf2/HO-1 signaling pathway, thus exerting the protection effects on CIRI.

## Conclusions

 Icariside II can alleviate the CIRI in rats through reducing brain tissue oxidative stress and improving Nrf2/HO-1 expression. This study has provided an experimental reference for the clinical application of icariside II to prevention and treatment of CIRI. 
